# Clinical significance of CDC25A and CDC25B expression in squamous cell carcinomas of the oesophagus

**DOI:** 10.1054/bjoc.2001.1934

**Published:** 2001-08

**Authors:** K Nishioka, Y Doki, H Shiozaki, H Yamamoto, S Tamura, T Yasuda, Y Fujiwara, M Yano, H Miyata, K Kishi, H Nakagawa, A Shamma, M Monden

**Affiliations:** Department of Surgery and Clinical Oncology, Graduate School of Medicine, Osaka University, 2-2-E2, Yamadaoka Suita, Osaka, 565-0871, Japan

**Keywords:** squamous cell carcinoma of the oesophagus, CDC25A, CDC25B, prognosis

## Abstract

CDC25A, CDC25B and CDC25C belong to a family of protein phosphatases which activate the cyclin-dependent kinase at different points of the cell cycle. According to accumulating evidence, CDC25A and CDC25B seem to possess oncogenic properties. We have analysed these expressions by immunohistochemistry, western blot and RT-PCR in a series of 100 patients with squamous cell carcinoma of the oesophagus. When compared with non-cancerous cells, CDC25A and CDC25B were strongly expressed in the cytoplasm of cancer cells, with positive (+) classification in 46% (46 cases) and 48% (48 cases), respectively. There was no significant correlation between CDC25A and CDC25B expression, nor was there any association with the expression of other cell cycle-regulating molecules, including cyclin D1, Rb, p16^INK4^, p27^KIP1^ and PCNA (proliferating cell nuclear antigen). CDC25A (+), as well as CDC25B (+), was more frequently found in patients with deeper tumour invasion and lymph node metastasis, while tumour size was correlated only with CDC25A expression. Postoperative survival was significantly poorer for CDC25A (+) patients than CDC25A (–) patients, but was not affected by the CDC25B status. Nuclear localization of CDC25A was observed in 51 cases (51%), regardless of its cytoplasmic expression, and was not associated with clinico-pathological factors or prognosis. Multivariate analysis revealed only the CDC25A status to be an independent significant prognostic factor among these biological and clinico-pathological factors. CDC25A but not CDC25B may be a new prognostic factor for squamous cell carcinoma of the oesophagus. Thus, regulation of the G1 checkpoint in the cell cycle may be important in oesophageal carcinogenesis, which may also involve many other oncogenes. © 2001 Cancer Research Campaign http://www.bjcancer.com


British Journal of Cancer (2001) **85**(3), 412–421
© 2001 Cancer Research Campaign
doi: 10.1054/ bjoc.2001.1934, available online at http://www.idealibrary.com on IDEAL®

http://www.bjcancer.com

### Clinical significance of CDC25A and CDC25B expression in squamous cell carcinomas of the oesophagus

**K Nishioka, Y Doki, H Shiozaki, H Yamamoto, S Tamura, T Yasuda, Y Fujiwara, M Yano, H Miyata, K Kishi, H Nakagawa, A Shamma and M Monden**

Department of Surgery and Clinical Oncology, Graduate School of Medicine, Osaka University 2-2-E2, Yamadaoka, Suita, Osaka, 565-0871, Japan

**Summary** CDC25A, CDC25B and CDC25C belong to a family of protein phosphatases which activate the cyclin-dependent kinase at different points of the cell cycle. According to accumulating evidence, CDC25A and CDC25B seem to possess oncogenic properties. We have analysed these expressions by immunohistochemistry, western blot and RT-PCR in a series of 100 patients with squamous cell carcinoma of the oesophagus. When compared with non-cancerous cells, CDC25A and CDC25B were strongly expressed in the cytoplasm of cancer cells, with positive (+) classification in 46% (46 cases) and 48% (48 cases), respectively. There was no significant correlation between CDC25A and CDC25B expression, nor was there any association with the expression of other cell cycle-regulating molecules, including cyclin D1, Rb, p16$^{INK4}$, p27$^{KIP1}$ and PCNA (proliferating cell nuclear antigen). CDC25A (+), as well as CDC25B (+), was more frequently found in patients with deeper tumour invasion and lymph node metastasis, while tumour size was correlated only with CDC25A expression. Postoperative survival was significantly poorer for CDC25A (+) patients than CDC25A (–) patients, but was not affected by the CDC25B status. Nuclear localization of CDC25A was observed in 51 cases (51%), regardless of its cytoplasmic expression, and was not associated with clinico-pathological factors or prognosis. Multivariate analysis revealed only the CDC25A status to be an independent significant prognostic factor among these biological and clinico-pathological factors. CDC25A but not CDC25B may be a new prognostic factor for squamous cell carcinoma of the oesophagus. Thus, regulation of the G1 checkpoint in the cell cycle may be important in oesophageal carcinogenesis, which may also involve many other oncogenes. © 2001 Cancer Research Campaign http://www.bjcancer.com

**Keywords**: squamous cell carcinoma of the oesophagus; CDC25A; CDC25B; prognosis

Cell cycle checkpoints are overcome by sequential activation of cyclin-dependent kinases (CDKs), which are regulated in several ways, including by binding with cyclins, sequestration of CDK inhibitors, and phosphorylation on the CDKs themselves (Hunter and Pines, 1994; Sherr, 1994). Phosphorylation on CDKs is functionally classified as stimulatory phosphorylation on the tyrosine residue by CAK (CDK-activating kinase) and inhibitory phosphorylation on threonine and tyrosine residues at the ATP binding site. The latter is controlled by wee1 kinase and the CDC25 phosphatase family (Pines, 1995). Three members of the CDC25 family (CDC25A, B and C) are commonly characterized as cell cycle oscillators in different phases of the cell cycle, in which both CDC25B and CDC25C work at the G2/M checkpoint, and CDC25A at the G1 checkpoint (Galaktionov and Beach, 1991; Nagata et al, 1991; Sadhu et al, 1990). During carcinogenesis, both CDC25A and CDC25B are over-expressed in various human malignancies including non-Hodgkin's lymphoma, breast cancer, non-small cell lung cancer, and head and neck cancer, however, no alteration of CDC25C has yet been reported (Galaktionov et al, 1995; Gasparotto et al, 1997; Hernandez et al, 1998; Wu et al, 1998). The potentiality of being an oncogene has been experimentally demonstrated with CDC25A and CDC25B, which were shown to transform murine fibroblasts in cooperation with mutated Ha-ras or loss of Rb (retinoblastoma gene) (Galaktionov et al, 1995).

*Received 19 October 2000*
*Revised 6 March 2001*
*Accepted 27 April 2001*

*Correspondence to:* Y Doki

In addition, CDC25A is over-expressed in azoxymethane-induced murine colon cancer (Dixon et al, 1998), and transgenic mice over-expressing CDC25B show enhanced tumorgenicity on DMBA (9,10-dimethyl-1, 2-benzanthracene) treatment (Yao et al, 1999).

Disorders of the cell cycle and cell cycle-regulating molecules are characteristics of cancer cells. In squamous cell carcinoma of the oesophagus, such disorders are concentrated at the G1 checkpoint, where amplification of cyclin D1 and loss of Rb, p16$^{INK4}$ and p27$^{KIP1}$ are frequently observed. We have found disorders of these molecules in more than 80% of oesophageal SCCs (squamous cell carcinomas) (Shamma et al, 1998). Some of these disorders greatly affect the clinical outcome, independently of other clinico-pathological parameters, and have been found to be associated with sensitivity for chemotherapy and/or radiation therapy via induction of tumour cell apoptosis (Coco Martin et al, 1999; Fukuoka et al, 1996; Kokunai and Tamaki, 1999; Warenius et al, 1996).

In the present study, we investigated the implication of the presence of CDC25 phosphatases in human oesophageal cancers by immunohistochemistry and molecular biology, and found that CDC25A over-expression is more strongly associated with advanced clinical stage and poor patient prognosis than disorders of other cell-cycle regulating molecules.

### MATERIALS AND METHODS

### Patients and tissue samples

Surgical specimens were obtained from 100 patients (80 males and 20 females) who had squamous cell carcinoma of the oesophagus and underwent subtotal oesophagotomy with lymph node dissection at the Department of Surgery II, Osaka University Medical School between 1990 and 2000. The age of the patients ranged from 45 to 80 years (mean: 61.2 ± 7.6 years). None had received irradiation or chemotherapy before surgery nor had haematogenic metastases at the time of surgery. The resected surgical specimens were fixed in 10% formaldehyde, processed through graded ethanol, and embedded in paraffin. A piece of each tissue sample was immediately frozen in liquid nitrogen and stored at – 80˚C until use for analyses by RT-PCR (reverse transcription polymerase chain reaction) and immunoblotting.

### Antibodies

The following antibodies were used in this study: rabbit polyclonal anti-human CDC25A antibody (Santa Cruz Biotechnology, CA), mouse monoclonal anti-human CDC25B antibody (Transduction Laboratories, Lexington, KY), rabbit polyclonal anti-human cyclin D1 antibody, M-20 (Santa Cruz Biotechnology, CA), mouse monoclonal IgG against human Rb protein, G3-245 (Pharmingen, San Diego, CA), rabbit polyclonal anti-human $p16^{INK4}$ (anti-serum), Catalog No. 15126E (Pharmingen), rabbit polyclonal anti-human $p27^{KIP1}$ antibody, C-19 (Santa Cruz Biotechnology, CA), mouse monoclonal anti-human PCNA antibody, batch 107904 (Novacastra Laboratories, Newcastle, UK), mouse monoclonal anti-human HSP27 antibody (G3.1; StressGen Biotechnologies Corporation, Victoria, British Columbia, Canada) and mouse monoclonal anti-human beta-actin antibody, A5441 (SIGMA, St. Louis, MO). The final diluted concentrations were as follows: anti-CDC25A, 0.5 µg $ml^{-1}$; anti-CDC25B, 1.25 µg $ml^{-1}$; anti-cyclin D1, 0.5 µg $ml^{-1}$; anti-Rb protein, 5 µg $ml^{-1}$; anti-$p16^{INK4}$, 400-fold dilution of the anti-serum; anti-$p27^{KIP1}$, 2 µg $ml^{-1}$; anti-PCNA, 1µg $ml^{-1}$; anti-human HSP27, 1000-fold dilution and anti-human beta-actin, 5000-fold dilution. The lysate from Hela cells, obtained from Transduction Lab., was used as a positive control for CDC25B in western blot analysis (Gabrielli et al, 1996).

### Immunohistochemistry

Sections 4 µm thick were deparaffinized in xylene, rehydrated and boiled for antigen retrieval (Ciaparrone et al, 1998). Processes of immunohistochemistry for CDC25A and CDC25B were performed with a TeckMate Horizon automated staining system (DAKO) using a Vectastain ABC-peroxidase kit (Vector Labs, Burlingame, CA), as previously described (Okami et al, 1999). In the primary antibody reaction, the slides were incubated with appropriate antibodies for 1 h at room temperature. Those for cyclin D1, Rb, $p16^{INK4}$, and $p27^{KIP1}$ were previously described (Shamma et al, 1998).

### Immunohistochemical assessment of CDC25A and CDC25B

Assessment of the staining was performed by two independent observers (YD and KN) who had no knowledge of the tumour stage or patient history. The expressions of CDC25A and CDC25B were evaluated according to the frequency of positive staining in the cytoplasm and/or nucleus of cancer cells. Since positive staining of CDC25A was common but showed various frequencies in oesophageal cancers, its expression was classified as positive (+) in cases with more than 50% positive-stained cells, with other samples being classified as negative (–). Nuclear expression of CDC25A was evaluated and determined as positive when more than 10% of the cancer cells showed obvious nuclear staining. In the case of CDC25B expression, cases with more than 10% positive-stained cells were classified as positive (+) and others as negative (–). Evaluation criteria of cyclin D1, Rb, $p16^{INK4}$, and $p27^{KIP1}$ were previously described (Shamma et al, 1998).

### Western blot analysis for CDC25A and CDC25B

Approximately 100 mg of each sample was homogenized in 1 ml lysis buffer (50 mM Tris pH 8.0, 150 mM NaCl, 0.5% NP-40) with protease inhibitor (1 mM PMSF, 10 µg $ml^{-1}$ aprotinin, 10 µg $ml^{-1}$ leupeptin). The homogenate was centrifuged at 15 000 rpm for 20 min at 4˚C. The resulting supernatant was collected and the total protein concentration was determined by the Bradford protein assay (Bio Rad, CA).

Cell fractination was also performed for western blotting of CDC25A. Fifty mg of tissue sample was soaked in 500 µl of hypotonic buffer (10 mM HEPES, 1.5 mM $MgCl_2$, 10 mM KCl, 300 mM sucrose, 1 mM EDTA, 0.25 mM EGTA, 0.1 mM DTT, 1 mM PMSF, 100 µg $ml^{-1}$ leupeptin, 0.5% NP-40, pH 7.9) for 30 min and centrifuged at 15 000 rpm for 5 min at 4˚C. The supernatant was collected for cytoplasmic protein. The pellet was soaked in 300 µl hypertonic buffer (20 mM HEPES, 1.5 mM $MgCl_2$, 0.5 M NaCl, 25% Glycerol, 1 mM EDTA, 0.1 mM DTT, 1 mM PMSF, 100 µg $ml^{-1}$ leupeptin, 0.5% NP-40, pH 7.9) for 30 min and centrifuged at 15 000 rpm for 5 min at 4˚C. The supernatant was collected for nuclear protein. Each fraction protein concentration was determined as described above. Western blotting was performed, as described previously (Yamamoto et al, 1999). Briefly, 100 µg of the total protein was subjected to 10% polyacrylamaide gel electrophoresis (PAGE) followed by electroblotting onto a polyvinylidene difluoride (PVDF) membrane. After blocking in 5% skim milk, the membrane was incubated with 0.5 µg $ml^{-1}$ CDC25A or with 1 µg $ml^{-1}$ CDC25B antibody, followed by incubation with 0.5 µg $ml^{-1}$ of secondary antibody (anti-rabbit IgG horseradish peroxidase conjugate for CDC25A and anti-mouse IgG horseradish peroxidase conjugate for CDC25B, Promega Corp., Madison, WI). For detection of the immunocomplex, the ECL western blot detection system (Amersham, Aylesbury, UK) was used. An equal amount of protein from each tissue extract was confirmed by immunoblot for beta-actin and gel staining with Coomassie blue. HSP27, which is located only in the cytoplasm, served as a control for cell fractionation.

### RNA extraction and RT-PCR analysis

Total RNA was extracted with a single-step method using TRIZOL reagent (Life Technologies, Inc., Gaithersburg, MD) and complementary DNA (cDNA) was generated using avian myeloblastosis virus reverse transcriptase (Promega, Madison, WI), as previously described (Gabrielli et al, 1996). Briefly, 1 µg of RNA was incubated at 70˚C for 5 min and then put on ice before the addition of RT (reverse transcription) reaction reagents with oligo-(dT) 15 priming. The RT reaction was performed at 42˚C for 90 min, followed by heating at 95˚C for 5 min.

Semi-quantitative analysis for the expression of CDC25A or CDC25B mRNA was performed by the multiplex RT-PCR technique, using porphobilinogen deaminase (PBGD) (Chretien et al, 1988; Nagel et al, 1996) as the internal standard. To minimize the inter-PCR difference, PCR was performed with PBGD and CDC25A or CDC25B primers in identical tubes, under unsaturated conditions, as described previously (Okami et al, 1999).

© 2001 Cancer Research Campaign 

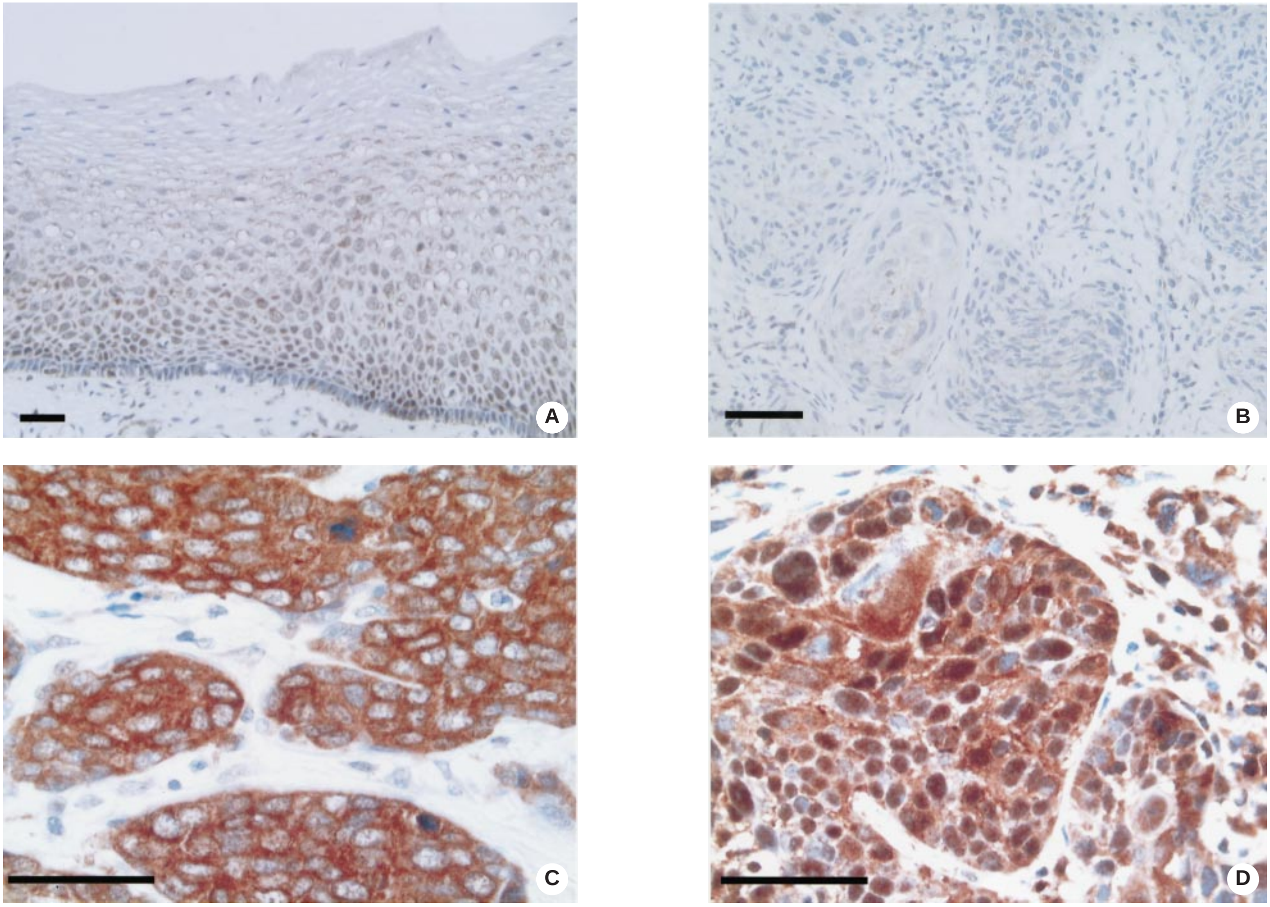

**Figure 1** Immunohistochemical staining of CDC25A in normal oesophageal epithelium (**A**) and squamous cell carcinomas of the oesophagus (**B, C, D**). Oesophageal cancers were classified as negative (**B**) and positive (**C, D**) according to the frequency of stained cells, and sometimes accompanied by nuclear CDC25A expression. (**D**) Original magnification × 100 (**A**) × 200 (**B**) and × 400 (**C**, **D**). Bars: 100 μm

PCRs were performed in a total volume of 25 μl reaction mixture containing 1 μl of cDNA template, 1X Perking Elmer PCR buffer, 1.5 mM $MgCl_2$, 0.8 mM deoxynucleotide triphosphates, 20 pmol of each primer for CDC25A or CDC25B, and 4 pmol each for PBGD, and 1 unit of Taq DNA Polymerase (AmpliTaq GoldTM, Roche Molecular Systems, Inc., NJ). The primer sets of CDC25A and CDC25B were designed to flank at least one intron and tested to ensure amplification of only cDNA to avoid amplification of any contaminating genomic DNA. We confirmed that the DNAs obtained from normal volunteers were absent of PCR products. The sequences of these PCR primers were as follows:

> CDC25A, (sense): 5′-GAGGAGTCTCACCTGGAAGTACA-3′ (NT 1297–1569 cDNA) and (antisense): 5′-GCCATTCAAAA CCAGATGCCATAA-3′. CDC25B, (sense): 5′-CACGCCCGT-GCAGAATAAGC-3′ (nt 1059–1475 cDNA) and (antisense): 5′-ATGACTCTCTTGTCCAGGCTACAGG-3′.

The primers for PBGD were synthesized as previously described (Nagel et al, 1996). The size of the amplicons for CDC25A, CDC25B, and PBGD were 272, 416, and 127 bp, respectively. The PCR conditions were as follows: initial denaturing at 95˚C for 12 min, followed by 35–40 cycles of 95˚C for 1 min, 62˚C for 1 min and 72˚C for 1 min, before a final extension at 72˚C for 10 min. A 10 μl portion of each PCR product was electrophoresed on 2% agarose gel, and stained with ethidium bromide. The PCR products were scanned by densitometry.

### Statistical analysis

Statistical analysis was performed using the Statview J-5.0 program (Abacus Concepts, Inc. Berkeley, CA). Twelve patients who underwent non-curative surgery with residual tumor (R2) (TNM classification, 1997), 8 patients who could not be followed during the postoperative follow-up and 10 patients who had undergone surgery within the prior 6 months, were excluded from survival analysis. For the remaining 70 patients, the follow-up period ranged from 6.1 months to 79.7 months (average 20.4 months). The Kaplan–Meier method was used to estimate death from oesophageal cancer and the log-rank test was used to estimate statistical significance. A Cox proportional hazards model was used to assess the risk ratio with simultaneous contribution from several covariates. The associations between the discrete variables were assessed using Fisher's exact test. Mean values were compared using the Mann-Whitney test. Differences causing $P$ values < 0.05 were accepted as statistically significant.

### RESULTS

### Immunohistochemical expression of CDC25A and CDC25B

In the non-cancerous stratified squamous epithelium of the oesophagus, CDC25A staining was weakly observed in the nuclei of

 *© 2001 Cancer Research Campaign*

**Table 1** Immunohistochemical expression of CDC25A and CDC25B in oesophageal cancers

| | Frequency of positively stained cells (%) | | | | |
|---|---|---|---|---|---|
| | 0–10 | 10–50 | 50–80 | 80–100 | Total |
| CDC25A | 11 | 43 | 38 | 8 | 100 |
| Nuclear CDC25A | 0 | 25 (49%) | 20 (39%) | 6 (12%) | 51 (100%) |
| CDC25B | 52 | 11 | 18 | 19 | 100 |

parabasal layer cells, while CDC25B was faintly detected in the cytoplasm of spinous layer cells (Figures 1A and 2A). In the interstitial tissue, both were weakly expressed in the germinal centre of lymph follicles. In most oesophageal cancer cells, CDC25A was strongly stained in the cytoplasm, sometimes accompanied by nuclear staining (Figures 1B, C and D). CDC25B was frequently observed to be strong in the cytoplasm of cancer cells, but not detectable in some tumours (Figures 2B and C). CDC25B expression was not apparent in the nuclei of cancer cells and was sometimes stronger in the deep invading cells than in the superficial cells (Figure 2D). The specificities of CDC25A and CDC25B staining were confirmed by an absorption test in which each antibody was mixed with an excess amount of antigen.

Expressions of CDC25A and CDC25B were evaluated according to the frequency of positive stained cells. As shown in Table 1, expression of CDC25A was more common than that of CDC25B. Therefore, CDC25A expression was classified as positive (+) in 46 cases (46%), in which more than 50% of the cells showed positive staining, while CDC25B expression was divided at the cut-off line of 10% positive-stained cells, resulting in 48 cases (48%) being judged CDC25B positive (+). Nuclear staining for CDC25A was observed in 51 cases (51%). However, the frequency of nuclear CDC25A expression was not correlated with that of cytoplasmic expression.

### Western blot and RT-PCR analysis for CDC25A and CDC25B expression

Western blot analyses for CDC25A and CDC25B protein (Figure 3) and cell fractionation for CDC25A subcellular localization (Figure 4) were performed using representative oesophageal cancer specimens with various immunostaining patterns,

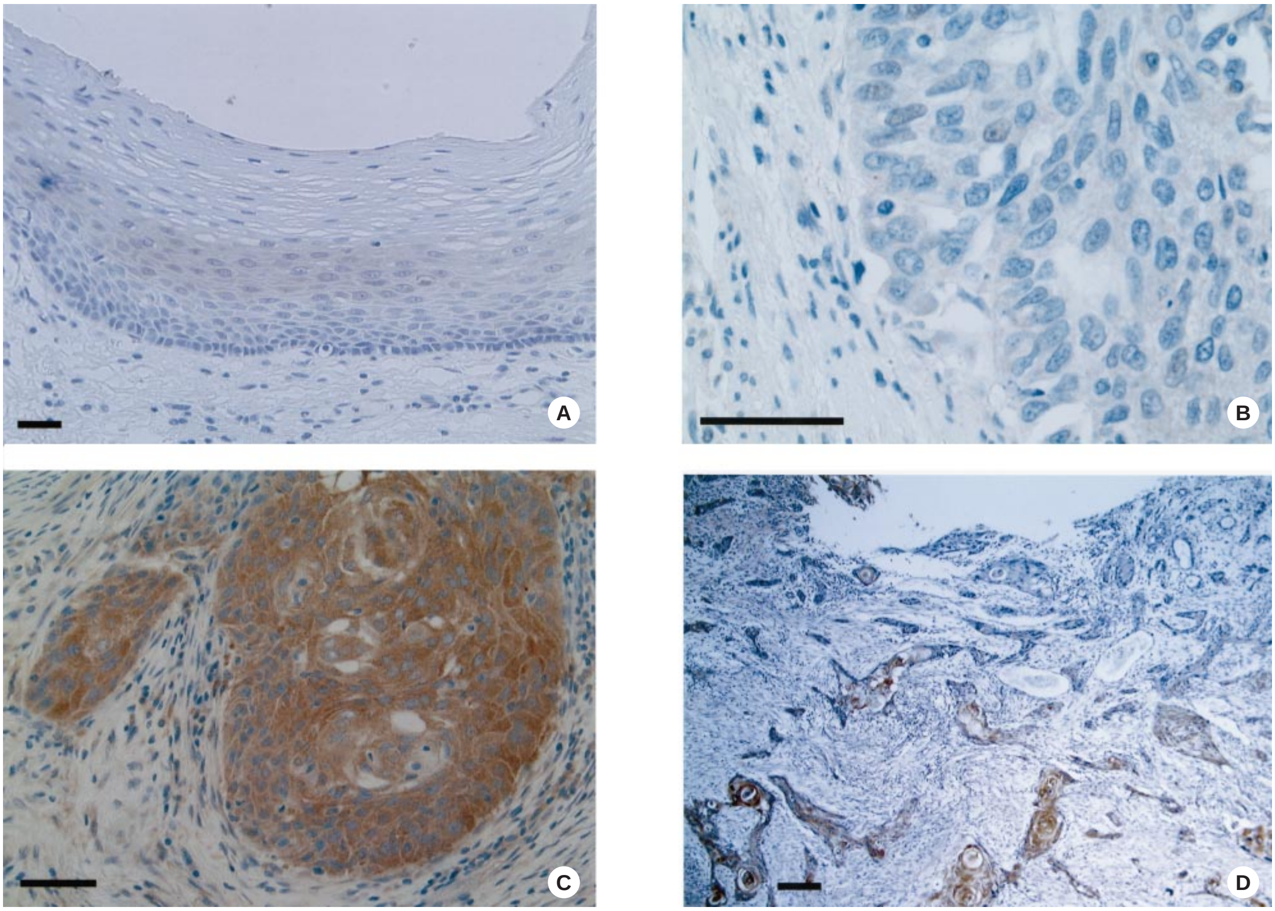

**Figure 2** Immunohistochemical staining of CDC25B in the normal oesophageal epithelium (**A**) and squamous cell carcinomas of the oesophagus (**B, C, D**). Oesophageal cancers were classified as negative (**B**) and positive (**C, D**) according to the frequency of stained cells. CDC25B expression was stronger in the deep invading cells than in the superficial cells (**D**). Original magnification × 100 (**A, D**) × 200 (**C**) and × 400 (**B**). Bars: 100 µm

© 2001 Cancer Research Campaign 

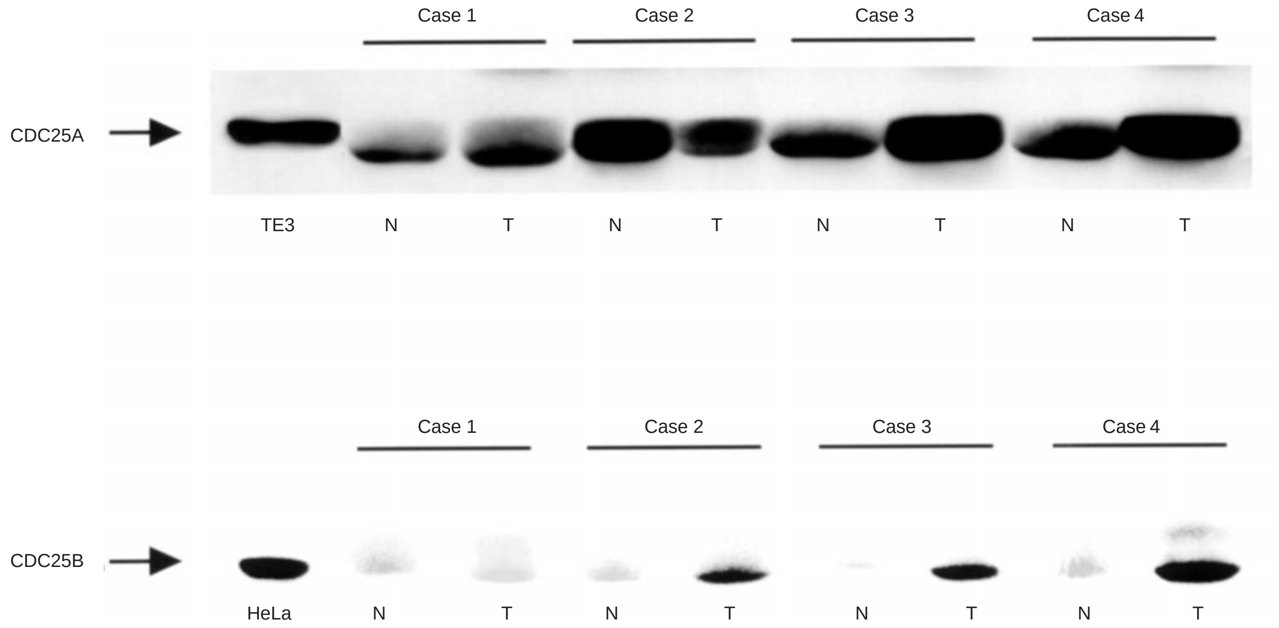

**Figure 3** Immunoblot analysis of CDC25A and CDC25B protein in oesophageal cancer tissues (T) and adjacent normal oesophageal epithelium (N). Arrows indicate the bands for CDC25A (58 kD) and CDC25B (63 kD). The amount of CDC25A in tumours is equivalent (case 1) or less (case 2), than that of normal mucosa, while it is more than twice the amount in case 3 and case 4, according to densitometry measurements. CDC25B was not expressed in normal mucosa or in the tumour tissue of case 1, while bands of CDC25B were apparent in the remaining tumour tissue samples. TE3, which expresses a high amount of CDC25A in both cytoplasm and nucleus, and Hela, which expresses CDC25B, served as positive controls. Evaluations by immunoblot were consistent with immunohistochemistry findings, led to negative (case 1 and case 2) and positive (case 3 and case 4) classifications for CDC25A and negative (case 1) and positive (case 2, case 3 and case 4) classifications for CDC25B. Nuclear staining for CDC25A was observed in case 4, but not in the others together with the corresponding non-cancerous mucosa. The bands at 63 kD, which were confirmed to be CDC25B both by positive control lysate of HeLa cells and by absorption tests using its blocking peptide, were observed only in some cancer tissues, and well correlated with their immunohistochemical expression (Figure 3). In contrast, the 58 kD CDC25A bands, which were also confirmed by the absorption tests, were ubiquitously observed among cancerous and non-cancerous tissues (Figure 3). CDC25A bands in oesophageal cancers were frequently stronger than those in non-cancerous mucosa, in agreement with the immunohistochemical evaluation results for CDC25A. In the subcellular localization analysis (Figure 4), normal mucosa mainly expressed CDC25A in the cytoplasm, however tumour tissues frequently expressed a high amount of CDC25A in the nuclear fraction, in agreement with the immunohistochemical evaluation results.

**Table 2** Relationship between CDC25s expression and clinico-pathological parameters

| | | **CDC25A** | | | **CDC25B** | | |
|---|---|---|---|---|---|---|---|
| | **Total** | **(+)** | **(–)** | ***P* value** | **(+)** | **(–)** | ***P* value** |
| Age (years) | | 63.2 ± 6.7 | 60.4 ± 7.8 | 0.1174 | 60.2 ± 7.5 | 62.5 ± 7.3 | 0.2059 |
| Gender | | | | | | | |
| Male | 80 | 40 | 40 | 0.1085 | 36 | 44 | 0.2298 |
| Female | 20 | 6 | 14 | | 12 | 8 | |
| Histological type | | | | | | | |
| G1, G2 | 61 | 25 | 36 | 0.2081 | 31 | 30 | 0.4803 |
| G3, G4 | 39 | 21 | 18 | | 17 | 22 | |
| Depth of invasion | | | | | | | |
| pT1, pT2 | 48 | 16 | 32 | 0.0141 | 17 | 31 | 0.0155 |
| pT3, pT4 | 52 | 30 | 22 | | 31 | 21 | |
| Nodal status | | | | | | | |
| pN0 | 44 | 13 | 31 | 0.0034 | 14 | 30 | 0.0041 |
| pN1 | 56 | 33 | 23 | | 34 | 22 | |
| TNM stage | | | | | | | |
| I II | 43 | 12 | 31 | 0.0016 | 14 | 29 | 0.0073 |
| III IV | 57 | 34 | 23 | | 34 | 23 | |
| Tumour size (mm) | | 67.8 ± 26.8 | 45.8 ± 22.6 | 0.0007 | 56.9 ± 20.7 | 51.9 ± 30.6 | 0.4515 |

 *© 2001 Cancer Research Campaign*

**Table 3** Relationship between CDC25A expression and other cell cycle regulators

| | CDC25A expression | | |
|---|---|---|---|
| | Positive | Negative | *P* value |
| CDC25B | | | |
| Positive | 25 | 23 | 0.2409 |
| Negative | 21 | 31 | |
| Cyclin D1* | | | |
| Positive | 16 | 18 | 0.9324 |
| Negative | 24 | 26 | |
| Rb* | | | |
| Positive | 32 | 33 | 0.5843 |
| Negative | 8 | 11 | |
| $p16^{INK4*}$ | | | |
| Positive | 17 | 22 | 0.4912 |
| Negative | 23 | 22 | |
| $p27^{KIP1*}$ | | | |
| Positive | 24 | 28 | 0.7318 |
| Negative | 16 | 16 | |
| PCNA* | | | |
| Positive | 18 | 26 | 0.1965 |
| Negative | 22 | 18 | |

*Eighty-four cases were available for the immunohistochemical evaluation of cyclin D1, Rb, $p16^{INK4}$, $p27^{KIP1}$, PCNA.

RT-PCR analyses for CDC25A and CDC25B were quantified by calculating the tumour/normal (T/N) ratio after adjustment with respect to the bands of PBGD, a housekeeping gene. In agreement with the immunoblot findings, the bands of CDC25A were recognized in all cancers and non-cancerous tissues, while PCR products for CDC25B were recognized only in some tumour samples, and not in non-cancerous mucosa (Figure 5). Three cases with T/N ratios of more than 3.5 for CDC25A (case 1, 2, 4) and three tumours with RT-PCR positive for CDC25B (case 2, 4, 5) consistently exhibited positive (+) immunostaining for CDC25A and CDC25B, respectively.

### Relationship of CDC25A and CDC25B expression with clinico-pathological factors and other cell cycle regulators

Table 2 summarizes the relationship between CDC25A and CDC25B expression and clinico-pathological factors. CDC25A (+) was more frequent in T3,4 (TNM classification, 1997) cases (30/52) than in T1,2 cases (16/48), and in patients with lymph node metastasis (33/56) than in those without it (13/44). Thus there was a strong positive correlation between CDC25A expression and depth of invasion ($P$ = 0.0141), nodal status ($P$ = 0.0034) and TNM stage ($P$ = 0.0016). CDC25B displayed the same relationship with depth of invasion ($P$ = 0.0155), nodal status ($P$ = 0.0041) and TNM stage ($P$ = 0.0073). Only CDC25A was correlated with tumour size, as 45.8 ± 22.6 mm and 67.8 ± 26.8 mm in negative and positive cases, respectively. Nuclear localization of CDC25A was not associated with any of the clinico-pathological factors (data not shown).

Table 3 shows the association of CDC25A with CDC25B and other cell cycle-regulating molecules, including cyclin D1, Rb, $p16^{INK4}$, $p27^{KIP1}$ and PCNA, which we have reported to be implicated in oesophageal carcinogenesis. There was no significant correlation between CDC25A and CDC25B expression, as well as other molecules. Also, there was no significant correlation between CDC25B and these cell cycle-regulating molecules (data not shown).

### Survival analysis

The cumulative postoperative survival curves revealed that patients with CDC25A (–) showed better prognosis than those with CDC25A (+) (5-year survival 66.2% vs 23.9%, $P$ = 0.0095) (Figure 6). However, the difference between CDC25B (–) and (+) (5-year survival 62.5 vs 18.7%) was not statistically significant ($P$ = 0.0755).

**Table 4** Prognostic factors in patients with squamous cell carcinoma of the oesophagus

| | Univariate analysis | | | Multivariate analysis | | |
|---|---|---|---|---|---|---|
| | RR | 95% CI | *P* value | RR | 95% CI | *P* value |
| CDC25A staining | | | | | | |
| (–) | 1 | | | 1 | | |
| (+) | 3.022 | 1.251–7.304 | 0.014 | 3.289 | 1.026–10.54 | 0.0451 |
| CDC25B staining | | | | | | |
| (–) | 1 | | | | | |
| (+) | 2.051 | 0.91–4.621 | 0.0831 | | | |
| Nodal status | | | | | | |
| pN0 | 1 | | | 1 | | |
| pN1 | 2.502 | 1.382–4.528 | 0.0024 | 0.584 | 0.139–2.447 | 0.4616 |
| Depth of invasion | | | | | | |
| pT1, pT2 | 1 | | | 1 | | |
| pT3, pT4 | 2.322 | 1.311–4.111 | 0.0038 | 2.901 | 0.757–11.124 | 0.1203 |
| Tumour size(mm) | | | | | | |
| 50 > | 1 | | | 1 | | |
| 50 ≤ | 2.752 | 1.087–6.966 | 0.0327 | 1.815 | 0.421–7.833 | 0.424 |
| $p27^{KIP1}$ | | | | | | |
| (+) | 1 | | | 1 | | |
| (–) | 2.017 | 1.144–3.555 | 0.0153 | 2.120 | 0.845–5.319 | 0.1094 |

RR; risk ratio, CI; confidence interval, (–); negative, (+); positive.

© 2001 Cancer Research Campaign 

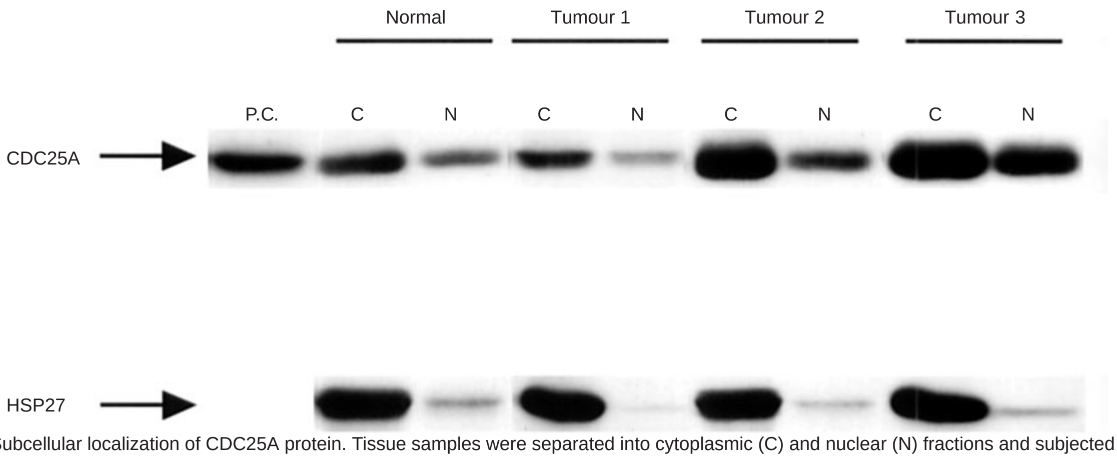

**Figure 4** Subcellular localization of CDC25A protein. Tissue samples were separated into cytoplasmic (C) and nuclear (N) fractions and subjected to immunoblotting of CDC25A. Recombinant CDC25A was used for positive control (PC), and HSP27, which is expressed mostly in cytoplasm, was used as the control for cell fractionation. Normal mucosa expressed CDC25A mainly in the cytoplasm, but tumour tissues expressed various amounts of nuclear CDC25A accompanied by cytoplasmic CDC25A. Immunohistochemical evaluation for CDC25A in each tumour gave results which were negative (Tumour 1), positive (Tumour 2), and positive with nuclear staining (Tumour 3)

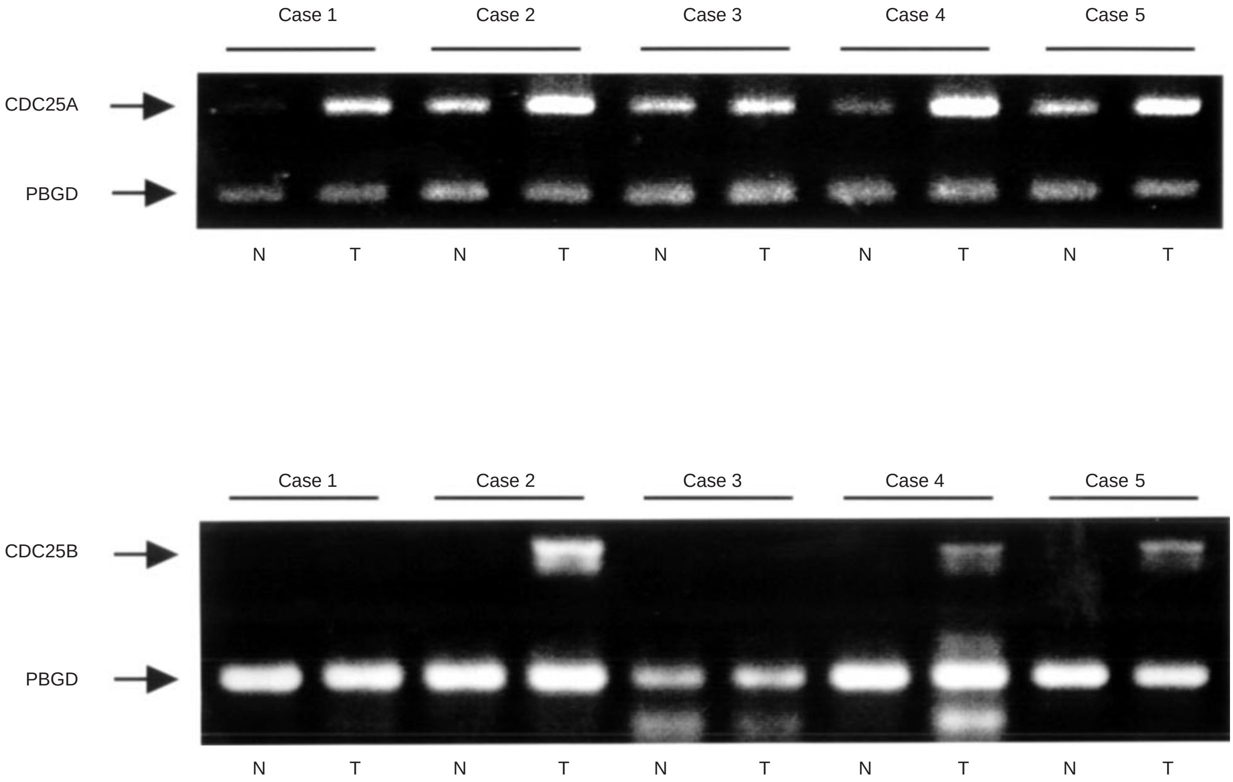

**Figure 5** Semiquantitative RT-PCR analysis of CDC25A and CDC25B m-RNA expression in oesophageal cancer tissues (T) and the adjacent normal oesophageal epithelium (N). The co-amplified PBGD gene served as an internal control. Immunohistochemical evaluation in each case was classified as negative (case 3 and case 5) and positive (case 1, case 2 and case 4) for CDC25A, and negative (case 1 and case 3) and positive (case 2, case 4 and case 5) for CDC25B

Using the Cox proportional hazard model, the depth of invasion, lymph node metastasis, TMN stage and $p27^{KJP1}$, which was revealed to be a significant prognostic factor in our previous study (Shamma et al, 1998), were found to be significant prognostic factors by univariate analysis (Table 4). Multivariate analysis revealed only CDC25A status to be an independent prognostic factor ($P = 0.0451$, risk ratio 3.289), with the others not being statistically significant. Nuclear localization did not affect postoperative survival (Figure 6).

### DISCUSSION

This is the first study to examine the expression of CDC25A and CDC25B in human oesophageal cancer tissues. We found over-expression of protein and mRNA of both CDC25A and CDC25B, and this is consistent with previous studies on head and neck cancers (Gasparotto et al, 1997) and non-small cell lung cancers (Wu et al, 1998).

 *© 2001 Cancer Research Campaign*

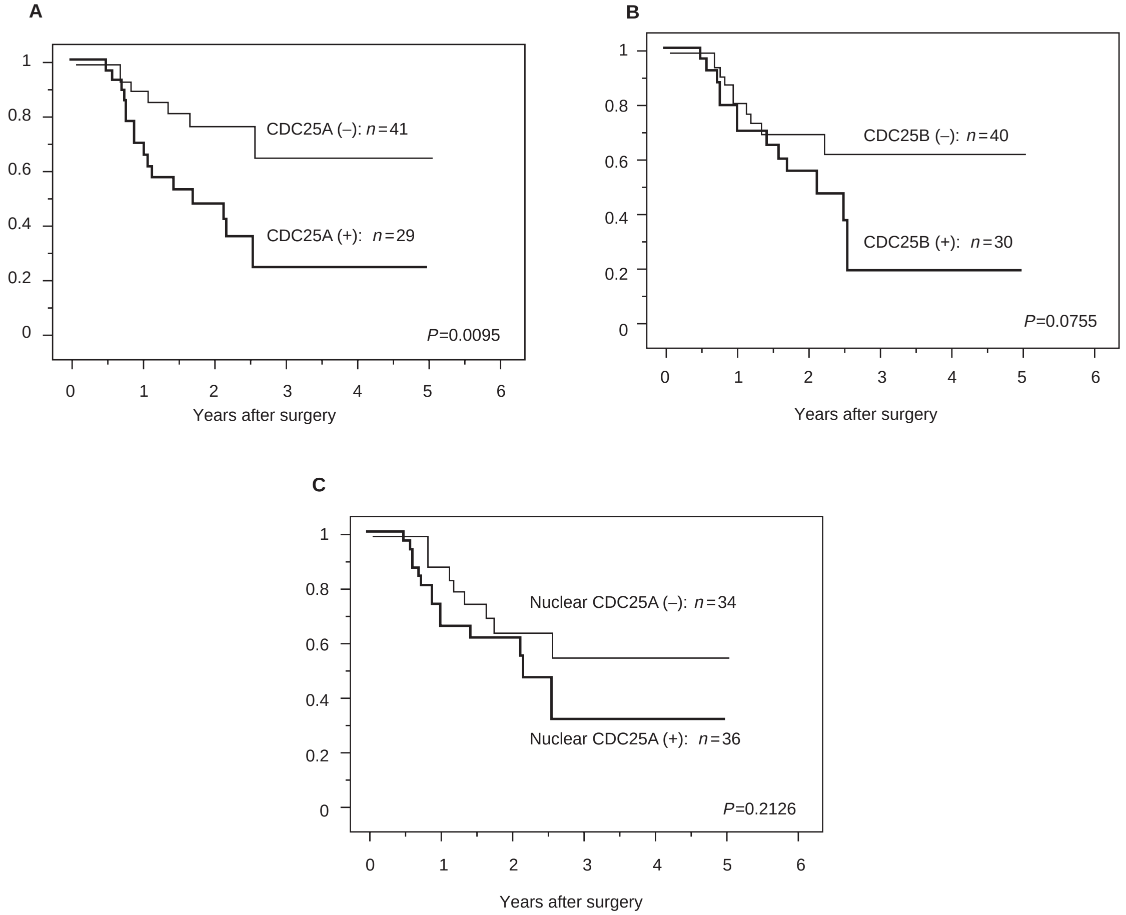

**Figure 6** Cumulative survival curves for oesophageal cancer patients classified according to CDC25A (**A**), CDC25B (**B**) and nuclear CDC25A (**C**) status (Kaplan–Meier method)

The CDC25A gene and CDC25B gene are located at 3p21 and 20p13, respectively, however, neither has been reported to be amplified in human oesophageal cancers. Moreover, although the latter locus is amplified in non-small cell lung cancers, it has not been associated with CDC25B over-expression (Wu et al, 1998). These findings suggest that not gene amplification but some transcriptional events are involved in protein and mRNA over-expression of CDC25s. One of the key molecules involved in transcription is c-myc, which strongly induces transcription of both CDC25A and CDC25B (Galaktionov et al, 1996) in cell experiments, and is frequently amplified in human oesophageal cancers (Lu et al, 1988) as well as other cancers in vivo. In non-Hodgkin lymphoma, the mRNA expression of CDC25B, but not CDC25A, is associated with c-myc overexpression (Hernandez et al, 1998). These findnds suggest that over-expression of CDC25B may have been induced by c-myc amplification in the present study for esophageal SCCs.

Comparison of CDC25A and CDC25B showed that over-expression of CDC25A is more frequent and ubiquitous, and that there is no correlation between their expressions. These findings are consistent with those of other studies, which simultaneously analysed CDC25A and CDC25B mRNA expression (Gasparotto et al, 1997; Kudo et al, 1997; Wu et al, 1998). CDC25A is expressed in a positive feedback manner during the G1/S phase as follows: CDC25A activates cyclin A/E-cdk2, resulting in release of E2F, which again induces CDC25A transcription (Chen and Prywes, 1999). Down-regulation by TGF-beta has been reported as another mechanism for CDC25A over-expression in vivo (Kang et al, 1999). These phenomena suggest that different regulation systems are involved in CDC25A and CDC25B transcription.

In the CDC25 family, the catalytic domain in the carboxyl terminus is well preserved, but little homology is observed in the amino-terminus domain, which is thought to be the regulatory domain (Galaktionov et al, 1995; Nagata et al, 1991; Sadhu et al, 1990). This would cause a difference in the cellular localization of CDC25A and CDC25B. Since CDC25A has a nuclear localization signal in the N-terminus, nuclear staining is frequently observed. However, the nuclear expression of CDC25A is not always correlated with cytoplasmic expression and is not associated with clinico-pathological factors or postoperative prognosis. In the case of CDC25B, although its nuclear localization has been reported during the cell cycle in the cultured cell lines, it was not observed in human oesophageal cancers in this study nor in gastric cancers in a previous study by Kudo et al (Kudo et al, 1997). Also, in previous studies, we did not detect nuclear expression of CDC25B in colon cancers (Takemasa et al, 2000) and hepatocellular carcinomas (unpublished observation by Yamamoto et al). Since a nuclear export system is involved in CDC25B activation (Karlsson et al, 1999), the CDC2/cyclin B complex may be de-phosphorylated by CDC25B in the cytoplasm at the G2 phase, and thereafter be transferred to the nucleus in the mitotic phase.

*© 2001 Cancer Research Campaign*

Interestingly, although there was no correlation between CDC25A and CDC25B expression, both were correlated with tumour invasion and metastasis. Moreover, since CDC25A was also associated with tumour size, it may contribute to cancer progression via tumour proliferation. During the cell cycle, the G1 checkpoint is critical for oesophageal cancers, therefore not only CDC25A, but also other G1-regulating molecules, including cyclin D1, Rb, $p16^{INK4}$ and $p27^{KIP1}$, are implicated (Shamma et al, 1998). The expression of CDC25A was not associated with those of the other G1-regulating molecules. Also, CDC25A expression was associated with postoperative prognosis, and surprisingly, multivariate analysis revealed that it was the only independent prognostic factor among clinico-pathological factors, such as depth of invasion, lymph node metastasis and $p27^{KIP1}$ expression. Recently, competitive interaction between CDC25A and $p21^{cip1}$ to cyclin-cdk complex has been demonstrated (Partha et al, 1997). However, in our preliminary study, $p21^{cip1}$ expression was more strongly affected by the status of both p53 and tumour differentiation than that of CDC25A (data not shown). It would be of interest to investigate the relationship between $p21^{cip1}$ and CDC25A in other cancers.

The effect of CDC25B status on postoperative survival was not statistically significant. Theoretically, CDC25B over-expression accelerates cell proliferation, and therefore would be a poor prognostic indicator. Recently, we found that CDC25B over-expression is associated with a high sensitivity for chemoradiation therapy through G2/M arrest (Miyata et al, 2000). Postoperative adjuvant therapy, including chemotherapy and radiation therapy, was performed for 26 patients of this series. We found no significant results for the clinical benefit of adjuvant therapy in this small number of patients, however, with a larger cohort, some influence of CDC25B status may be found.

In the present study, we used different cut-off lines for CDC25A (50%) and CDC25B (10%). When we used other cut-off lines for the data in Table 1, such as 10% or 80% for CDC25A or 50% or 80% for CDC25B, the differences in postoperative survival were smaller and not statistically significant, although the trend was the same. The cut-off lines in this study well reflect the biological properties of the molecules. The other cut-off lines led to biased separations, in which one side included too few cases to allow statisticaly significant differences.

We have presented here the significance of CDC25A as a novel prognostic factor in human oesophageal cancers. This study is a start toward elucidating the implication of CDC25s in clinical cancer treatment.

### ACKNOWLEDGEMENTS

This work was supported in part by a Grant-in-Aid for Scientific Research (B) (No. 12671223) and grants for Cancer Research (No. 12213078) (to H.Y.) from the Ministry of Education, Science, Sports and Culture.

### REFERENCES

Chen X and Prywes R (1999) Serum-induced expression of cdc25A gene by relief of E2F-mediated repression. *Mol Cell Biol* **19**: 4695–4702

Chomczynski P and Sacchi N (1987) Single-step method RNA isolation by acid guanidiniumthiocyanate-phenol-chloroform extraction. *Anal Biochem* **162**: 156–159

Chretien S, Dubart A, Beaupain D, Raich N, Grandchamp B, Rosa J, Goossens M and Romeo PH (1988) Alternative transcription and splicing of the human porphobilinogen deaminase gene result either in tissue-specific or in housekeeping expression. *Proc Natl Acad Sci USA* **85**: 6–10

Ciaparrone M, Yamamoto H, Yao Y, Sgambato A, Cattoretti G, Tomita N, Monden M, Rotterdam H and Weinstein I (1998) Localization and expression of p27KIP1 in multistage colorectal carcinogenesis. *Cancer Res* **58**: 114–122

Coco Martin JM, Balkenende A, Verschoo T, Lallemand F and Michalides R (1999) Cyclin D1 overexpression enhances radiation-induced apoptosis and radiosensitivity in a breast tumor cell line. *Cancer Res* **59**: 1134–1140

Dixon D, Moyana T and King MJ (1998) Elevated expression of the cdc25A protein phosphatase in colon cancer. *Exp-Cell-Res* **240**: 236–243

Fukuoka K, Shindoh M, Yamashita T, Fujinaga K, Amemia A and Totsuka Y (1996) High-risk HPV-positive human cancer cell lines show different sensitivity to cisplatin-induced apoptosis corelated with the p21Waf/Cip1 level. *Cancer Lett* **108**: 15–23

Galaktionov K and Beach D (1991) Specific activation of cdc25 tyrosine phosphatases by B-type cyclins: evidence for multiple roles of mitotic cyclins. *Cell* **67**: 1181–1194

Galaktionov K, Lee AK, Eckstein J, Draetta G, Meckler J, Loda M and Beach D (1995) CDC25 phosphatases as potential human oncogenes. *Science* **269**: 1575–1577

Galaktionov K, Chen X and Beach D (1996) Cdc25 cell-cycle phosphatase as a target of c-myc. *Nature (Lond)* **382**: 511–517

Gabrielli BG, De Souza CP, Tonks ID, Clark JM, Hayward NK and Ellem KA (1996) Cytoplasmic accumulation of cdc25B phosphatase in mitosis triggerrs centrosomal microtubule nucleation in HeLa cells. *J Cell Sci* **109**: 1081–1093

Gasparotto D, Maestro R, Piccinin S, Vukosavljevic T, Barzan L, Sulfaro S and Boiocchi M (1997) Overexpression of CDC25A and CDC25B in head and neck cancers. *Cancer Res* **57**: 2366–2368

Hernandez S, Hernandez L, Bea S, Cazorla M, Fernandez PL, Nadal A, Muntane J, Mallofre C, Montserrat E, Cardesa A and Campo E (1998) cdc25 cell cycle-activating phosphatases and c-myc expression in human non-Hodgikin lymphomas. *Cancer Res* **58**: 1762–1767

Hunter T and Pines J (1994) Cyclins and cancer. II: Cyclin D and CDK inhibitors come of age. *Cell* **79**: 573–582

Kang SH, Bang YJ, Jong HS, Kim NK and Kim SJ (1999) Rapid induction of p21 Waf1 but delayed down regulation of cdc25A in the TGF-beta-induced cell cycle arrest of gastric carcinoma cells. *Br J Cancer* **80**: 1144–1149

Karlsson C, Katich S, Hagting A, Hoffmann I and Pines J (1999) Cdc25B and cdc25C differ markedly in their properties as initiators mitosis. *J Cell Biol* **3**: 573–584

Kokunai T and Tamaki N (1999) Relationship between expression of p21Waf/Cip1 and radioresistance in human gliomas. *Jpn J Cancer Res* **90**: 638–646

Kudo Y, Yasui W, Ue T, Yamamoto S, Yokozaki H, Nikai H and Tahara E (1997) Overexpression of cyclin-dependent kinase-activating cdc25B phosphatase in human gastric carcinomas. *Jpn J Cancer Res* **88**: 947–952

Lu SH, Hsieh LL, Luo FC and Weinstein IB (1988) Amplification of the EGF receptor and c-myc genes in human esophageal cancers. *Int J Cancer* **42**: 502–505

Miyata H, Doki Y, Hitoshi S, Inoue M, Yano M, Fujiwara Y, Yamamoto H, Nishioka K, Kentaro K and Monden M (2000) CDC25B and p53 are independently implicated in radiation sensitivity for human esophageal cancers. *Clin Cancer Res* **6**: 4859–4865

Nagata A, Igarashi M, Junno S, Suto K and Okayama H (1991) An additional homolog of fission yeast cdc25+ gene occurs in humans and is highly expressed in some cancer cells. *New Biol* **3**: 959–968

Nagel S, Schmidt S, Thiede C, Huhn D and Neubauer A (1996) Quantification of Bcr-Abl transcripts in chronic myelogenous leukemia (CML) using standardized, internally controlled, competitive differential PCR(CD-PCR). *Nucleic Acids Res* **24**: 4102–4103

Okami J, Yamamoto H, Fujiwara Y, Tsujie M, Kondo M, Noura S, Oshima S, Nagano H, Dono K, Umeshita K, Ishikawa O, Sakon M, Matsuura N and Monden M (1999) Overexpression of cyclooxygenase-2 in carcinoma of the pancreas. *Clin Cancer Res* **5**: 2018–2024

Pines J (1995) Cyclins and cyclin-dependent kinasees: theme and variations. *Adv Cancer Res* **66**: 181–212

Sadhu K, Reed SI, Richardson H and Russell P (1990) Human homolog of fission yeast cdc25 mitotic inducer is predominantly expressed in G2. *Proc Natl Acad Sci USA* **87**: 5139–5143

Saha P, Eichbaum Q, Silberman ED, Mayer B and Dutta A (1997) $p21^{CIP1}$ and Cdc25A: Competition between an Inhibitor and an Activator of Cyclin-Dependent Kinases *Mol Cell Biol* **17**: 4338–4345

Shamma A, Doki Y, Shiozaki H, Tsujinaka T, Inoue M, Yano M, Kimura Y, Yamamoto M and Monden M (1998) Effect of cyclin D1 and associated

© 2001 Cancer Research Campaign proteins on proliferation of esophageal squamous cell carcinoma. *Int J Oncology* **13**: 455–460
Sherr CJ (1994) Mammalian G1 cyclins. *Cell* **79**: 551–555
Takemasa I, Yamamoto H, Tomita N, Sekimoto M, Ohue M, Noura S, Miyake Y, Aihara T, Tamaki Y, Sakita I, Matsuura N and Monden M (2000) Assessment for increased expression of cdc25B phosphatase as a novel prognostic marker in human colorectal carcinomas. *Cancer Res* **60**: 3043–3050
TNM classification by UICC (fifth edition, 1997)
Warenius HM, Seabra LA and Maw P (1996) Sensitivity to cis-biamminedichloroplatinum in human cancer cells is related to expression of cyclin D1 but not c-raf-1 protein. *Int J Cancer* **67**: 224–231
Wu W, Fan YH, Kemp BL, Walsh G and Mao L (1998) Overexpression of cdc25A and cdc25B is frequent in primary non-small cell lung cancer but is not associated with overexpression of c-myc. *Cancer Res* **58**: 4082–4085
Yamamoto H, Soh J-W, Shirin H, Xing W-Q, Lim JT, Yao Y, Slosberg E, Tomita N, Schieren I and Weinstein IB (1999) Comparative effects of overexpression of p27KIP1 and p21CIP1/WAFT on growth and differentiation in human colon carcinoma cells. *Oncogene* **18**: 103–115
Yao Y, Slosberg E, Wang L, Hibshoosh H, Zhang Y, Xing W and Weinstein B (1999) Increased susceptibility to carcinogen-induced mammary tumors in MMTV-Cdc25B transgenic mice. *Oncogene* **18**: 5159–5166

*© 2001 Cancer Research Campaign*
